# Symbiotic *Wolbachia* in mosquitoes and its role in reducing the transmission of mosquito-borne diseases: updates and prospects

**DOI:** 10.3389/fmicb.2023.1267832

**Published:** 2023-10-13

**Authors:** Awoke Minwuyelet, Giulio Petronio Petronio, Delenasaw Yewhalaw, Andrea Sciarretta, Irene Magnifico, Daria Nicolosi, Roberto Di Marco, Getnet Atenafu

**Affiliations:** ^1^Department of Biology, College of Natural and Computational Sciences, Debre Markos University, Debre Markos, Ethiopia; ^2^Department of Medicine and Health Sciences, University of Molise, Campobasso, Italy; ^3^Tropical and Infectious Diseases Research Center, Jimma University, Jimma, Ethiopia; ^4^Faculty of Health Sciences, School of Medical Laboratory Sciences, Jimma University, Jimma, Ethiopia; ^5^Department of Agriculture, Environment and Food Sciences, Università degli Studi del Molise, Campobasso, Italy; ^6^Department of Pharmaceutical and Health Sciences, Università degli Studi di Catania, Catania, Italy

**Keywords:** mosquito symbiont, *Wolbachia*, *Aedes*, *Anopheles*, *Culex*, mosquito, mosquito-borne diseases

## Abstract

Mosquito-borne diseases such as malaria, dengue fever, West Nile virus, chikungunya, Zika fever, and filariasis have the greatest health and economic impact. These mosquito-borne diseases are a major cause of morbidity and mortality in tropical and sub-tropical areas. Due to the lack of effective vector containment strategies, the prevalence and severity of these diseases are increasing in endemic regions. Nowadays, mosquito infection by the endosymbiotic *Wolbachia* represents a promising new bio-control strategy. Wild-infected mosquitoes had been developing cytoplasmic incompatibility (CI), phenotypic alterations, and nutrition competition with pathogens. These reduce adult vector lifespan, interfere with reproduction, inhibit other pathogen growth in the vector, and increase insecticide susceptibility of the vector. Wild, uninfected mosquitoes can also establish stable infections through trans-infection and have the advantage of adaptability through pathogen defense, thereby selectively infecting uninfected mosquitoes and spreading to the entire population. This review aimed to evaluate the role of the *Wolbachia* symbiont with the mosquitoes (*Aedes, Anopheles,* and *Culex*) in reducing mosquito-borne diseases. Global databases such as PubMed, Web of Sciences, Scopus, and pro-Quest were accessed to search for potentially relevant articles. We used keywords: *Wolbachia*, *Anopheles*, *Aedes*, *Culex*, and mosquito were used alone or in combination during the literature search. Data were extracted from 56 articles’ texts, figures, and tables of the included article.

## 1. Introduction

Due to their high adaptation capacity to various environments, mosquitoes have endured for millions of years (Couper et al., 2021). Different pathogenic, endosymbiont and symbiotic organisms have the ability to infect them. The main carriers of human pathogens are various species of mosquitoes from the genera *Aedes, Anopheles,* and *Culex.* Those mosquito genera are vectors of emerging and reemerging human diseases caused by pathogens, such as protozoan parasites, viruses, and nematodes (Iturbe-Ormaetxe et al., 2011).

Among the protozoan parasitic diseases, malaria is caused by different *Plasmodium* species. It is a life-threatening disease spread to humans by the bite of infected female *Anopheles* mosquitoes. According to the *World Health Organization* (WHO) report in 2022, globally, more than 3.2 billion people (almost half the world’s population) are at risk of malaria; furthermore, 245 million malaria cases have been recorded, with a mortality of 0.6 million. Children are the most affected group of patients. Malaria is also a great burden from an economic point of view; $ 12 billion is lost per year in economic productivity in Africa alone (WHO, 2022).

Similarly, among mosquito-borne viral diseases, viruses belonging to the *Flaviviridae* family, such as *Dengue* virus, *Zika* virus, yellow fever virus, chikungunya virus, and *West Nile* virus, can be transmitted to humans by *Aedes aegypti* and *Ae. albopictus*.

About half of the world’s population is at risk of dengue, which is estimated to infect 100–400 million people yearly. It is found in tropical and subtropical climates worldwide, mainly in urban and semi-urban areas (Leta et al., 2018). Likewise, West Nile fever is caused by an RNA virus, namely *West Nile* virus (WNV). The virus causes severe disease in birds, horses, and other mammals, but most human infections occur through the bite of infected mosquitoes. About 1 in 150 infected people develop neurological disease and die. It is common in Africa, Europe, the Middle East, North America, and Western Asia (Leta et al., 2018; CDC, 2023). In addition, Yellow fever is caused by an arbovirus and is transmitted to humans through the bites of infected *Aedes* and *Haemagogus* mosquitoes. It is a high-impact high-threat disease with the risk of cross-boundary transmission (Leta et al., 2018; WHO, 2023a).

Moreover, the Zika virus is transmitted to humans through the bites of infected mosquitoes, mainly *Ae. aegypti*, particularly in tropical regions. Zika virus infection clinical manifestation is similar to other arboviruses, with fever, skin rash, conjunctivitis, muscle and joint pain, fatigue, and headache (Leta et al., 2018; WHO, 2023b). On the other hand, Chikungunya fever is caused by an RNA virus belonging to the *alphavirus* genus, the *Togaviridae* family. Infection in humans occurs through the bite of infected female mosquitoes (commonly *Ae. aegypti* and *Ae. albopictus*). More than 2 million cases arise each year. The disease is now identified in more than 110 countries (Bettis et al., 2022).

Among nematode infections transmitted by mosquito vectors, filariasis is mainly caused by the filarial worm *Wuchereria bancrofti,* and less commonly *Brugia malayi* and *Brugia timori. Anopheles* is the main filariasis vector in Africa, however, in the Americas the main vector is *Culex*. It is also transmitted by the bite of infected *Aedes* and *Mansonia* species. Filariasis has been considered a neglected tropical disease. However, it is the second leading cause of permanent malformation and disability, next to leprosy worldwide. Lymphatic filariasis affects the lymphatic system and causes abnormal enlargement of body parts, which can cause pain, severe disability, and social stigma. It affects more than 120 million of people in 72 tropical and subtropical countries. Over 882 million people in 44 countries worldwide remain threatened by lymphatic filariasis and require preventive chemotherapy to stop the spread of this parasitic infection (Bizhani et al., 2021; WHO, 2021).

**Figure 1:**
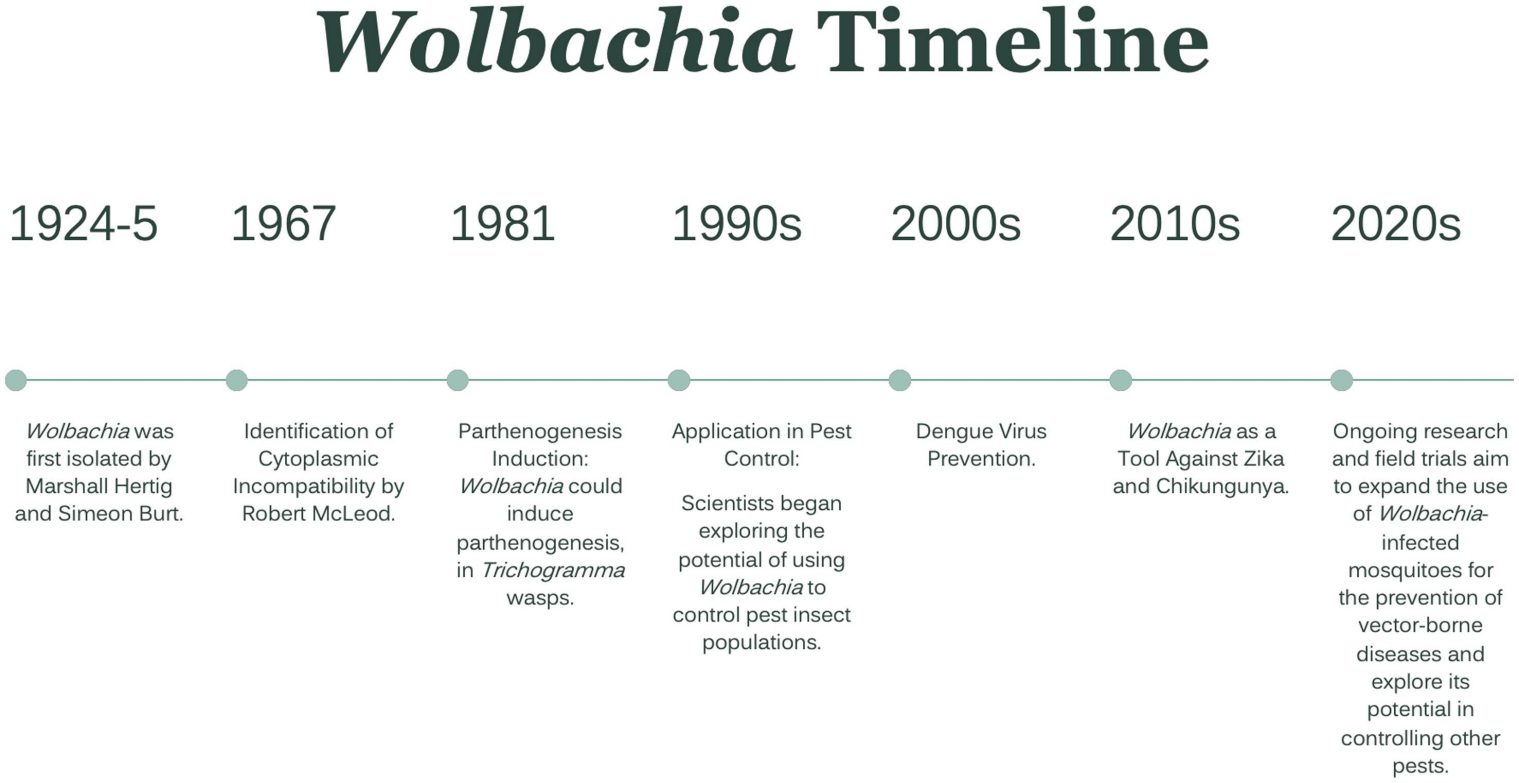
*Wolbachia* Timeline. From first isolation to ongoing research.

To reduce the threat and burden of these vector-borne diseases, insecticides have been widely used in the last many years. However, due to the frequent and prolonged use of insecticides to control insect disease vectors and pests of crops, mosquitoes developed resistance to several classes of insecticides. As a result, bacteria belonging to the *Wolbachia* genus have been proposed as potential candidates for mosquito-borne disease control strategies (van den Berg et al., 2021). A brief timeline of *Wolbachia* isolation, the impact of infection, and utilization as a prevention method is presented in Figure 1 (Werren and O’Neill, 1997; Carrington et al., 2011; Kamtchum-Tatuene et al., 2017; Dorigatti et al., 2018).

*Wolbachia* is a genus of Gram-negative, non-spore-forming, obligate intracellular parasitic bacteria that frequently infect mosquitoes. It is a member of the Alphaproteobacteria belonging to the Rickettsiales order. The bacterium was first isolated in 1924 by Hertig and Wolbach from the *Cx. pipiens* germlines (Hertig and Wolbach, 1924). Later in 1936, Hertig, designated it as *Wolbachia pipientis* (Hertig, 1936; Philip, 1956).

**Figure 2:**
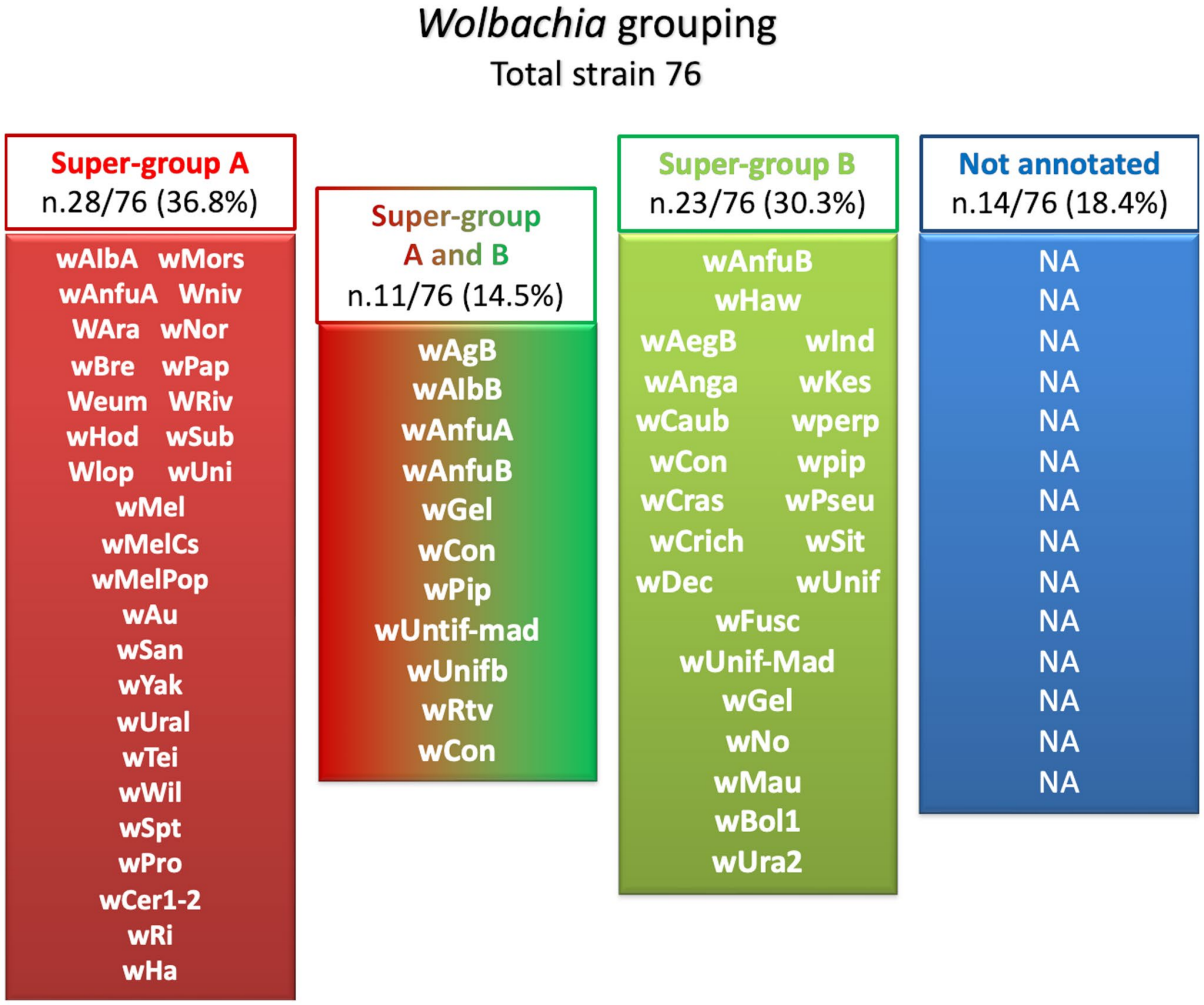
*Wolbachia* strains isolated from mosquito grouping. Of the 76 isolated *Wolbachia* strains, 28 (36.8%) belong to super-group A (Red), 23 strains (30.3%) are also grouped in super-group B (Green), 11 strains (14.5%) are also grouped under both A and B (Red and Green), while 14 (18.4%) were non annotated (Blue).

In the last two decades, different strains of *Wolbachia* were isolated and identified by genome sequencing: *Wolbachia* wAna, *Wolbachia* wSim, *Wolbachia* wMel, and *Wolbachia* wMoj from *Drosophila* species (Salzberg et al., 2005). Then different *Wolbachia* strains are grouped into two major phylogenetic lineages. More than 18 clades, ranging from A to R, have been identified, and almost all were isolated from arthropods (Landmann, 2019). The general distribution of *Wolbachia* strains and their associated supergroups in mosquitoes are summarized in Figure 2 (Inácio da Silva et al., 2021).

Besides mosquitoes, these intracellular bacteria selectively infect arthropods, nematodes, and other organisms but are harmless to humans (Osei-Poku et al., 2012). It forms endosymbiotic relationships that range from parasitism to mutualism (Zug and Hammerstein, 2012; Sullivan, 2017). Parasitism and mutualism are host, environment, temperature, and density-dependent induced by the same genetic machinery and shifted by selection (Bordenstein et al., 2009; Zug and Hammerstein, 2015; Rohrscheib et al., 2016). Parasitism persistently affects several hosts’ biological indicators, such as physiology, immunity, and host development (Werren et al., 2008; Gutzwiller et al., 2015). The host’s capacity for reproduction was also altered. Additionally, it makes arthropods sterile, and infertile, with reduced longevity, which strongly impacts male mosquitoes (Werren and Windsor, 2000; Ahmed et al., 2015; Sicard et al., 2019). On the other hand, mutualism provides resistance to viral pathogens or the provision of metabolites during host nutritional stress (Allman et al., 2020; Kaur et al., 2021).

The bacterium has the ability to be transmitted vertically through insect eggs and spread horizontally across populations (Hedges et al., 2008; Zug and Hammerstein, 2012; Duron et al., 2015). A vertically transmitted *Wolbachia* is frequently found in the insect’s endosymbionts, with a 28%–30% prevalence of naturally infected mosquitoes (Kittayapong et al., 2000; Dorigatti et al., 2018; Inácio da Silva et al., 2021). Furthermore, there are different types of symbiont transmission, from vertical (genetic) to horizontal (infectious), with horizontal transmission opting for parasitism. In contrast, vertically transmitted endosymbionts evolve toward reciprocity (Zug and Hammerstein, 2015) among naturally infected genera: *Aedes, Culex*, *Drosophila,* and other insect species (Osei-Poku et al., 2012; Sicard et al., 2019; Inácio da Silva et al., 2021) but not commonly reported in *Ae. aegypti* and *Anopheles* species.

Even though *Wolbachia* infection is transmitted between unrelated species, it spreads more quickly among related species. As a result, strains that naturally exist in mosquitoes are suitable for trans-infection into different vector species, enabling bacterial diffusion among mosquito populations (Turelli and Hoffmann, 1991).

*Wolbachia*-infected mosquitoes reduce mosquito-borne diseases by reducing competent mosquito populations or the vector’s number of mosquitoes and/or pathogen replication (Yen and Failloux, 2020). This is due to CI stimulated by the dynamics of *Wolbachia* strains introduced into a mosquito population and immune modulation (Kambris et al., 2010), which are triggered to change the host’s behavior and the pathogenic transmission effect (Sinkins et al., 2005; Hedges et al., 2008; Kambris et al., 2009, 2010; Dorigatti et al., 2018). This phenomenon reduces pathogen replication and disease transmissions by vectors to humans and/or animals.

In this review, we focused on assessing the role of *Wolbachia* infection in the genera *Aedes, Anopheles,* and *Culex* in reducing vector-borne diseases. Global electronic databases (PubMed, Web of Sciences, Scopus, and Pro-Quest) were used to search potentially relevant and most recent articles published from 2000 to 2022. *Wolbachia, Anopheles, Aedes, Culex*, and mosquito were used alone or in combination as a keyword during the literature search. The search was conducted from November 15th to December 12th, 2022. Papers were chosen according to topic pertinence; only research articles published in English and articles with all the required information were included in the review. Data were extracted from the included articles’ texts, figures, and tables, of the included articles. Preliminary 946 articles were accessed, of which only 56 were used for review, as shown in Supplementary Figure S1 and Supplementary Table S1.

## 2. *Wolbachia* strain and mosquito infection

This study retrieved 56 original studies. Of these, 32 and 13 studies reported infection of *Aedes* and *Anopheles* species by *Wolbachia*. Other 11 original studies revealed infection in *Culex* species (Supplementary Figure S1 and Supplementary Table S1).

### 2.1. *Wolbachia* infection in *Aedes* species

The genus *Aedes* includes more than 950 species and is one of the most widespread mosquito genera in the world (Rogers, 2023). Among them, *Ae. aegypti* and *Ae. albopictus* are the most known biological vectors of vector-borne diseases (Brelsfoard and Dobson, 2011; Silva et al., 2017; Damiani et al., 2022) and are included in this study. These two main species are primarily responsible for spreading filariasis, dengue, yellow fever, chikungunya, West Nile Virus, and Zika fever, which can result in serious human diseases (Hoey, 2000; Brasil et al., 2016). These illnesses are a major public health problem resulting in millions of infections and thousands of fatalities yearly (Caragata et al., 2021).

Due to the disease’s severity and the limitation of current prevention patterns, entomopathogenic bacteria have been explored to enhance current control measures and proposed as an effective strategy to reduce the increasing problem of vector-borne diseases (Turley et al., 2009; Mohanty et al., 2016; Yen and Failloux, 2020).

A recent molecular study by Li et al. (2023) in the Chinese province of Hainan revealed that the prevalence of *Wolbachia* was 86.7% from field-collected *Ae. albopictus* (Li et al., 2023). Another study conducted in eastern Thailand by Kittayapong et al. (2002) demonstrated the maternal transmission of *Wolbachia* from field-collected *Ae. albopictus* was nearly 100%. Wild infections also have efficient vertical transmission across host generations, essential for symbiosis. Even though there was no natural infection report of *Ae. aegypti* populations by *Wolbachia*, many studies indicated trans-infection techniques to infect non-wild infected mosquito populations (Turley et al., 2009; Walker et al., 2011; Jeffries and Walker, 2015; O’Neill, 2018; Ding et al., 2020; Liew et al., 2021). This technique established stable vertical transmission in *Ae. albopictus*, *Ae. aegypti* and *Anopheles* species (McMeniman et al., 2009; Iturbe-Ormaetxe et al., 2011; Calvitti et al., 2014).

#### 2.1.1. *Wolbachia* infection and its effect on *Aedes* species

**Figure 3:**
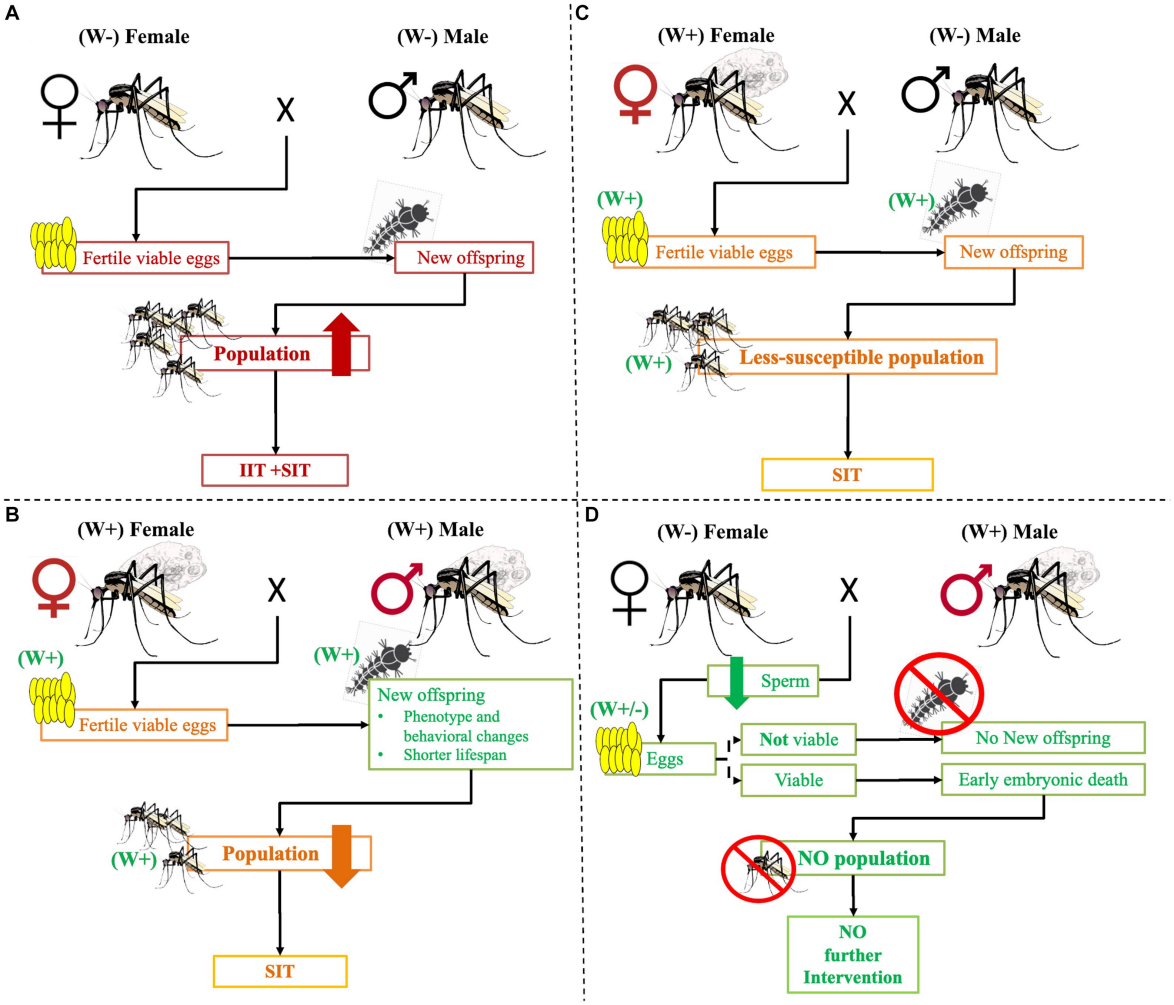
The possible crosses between *Wolbachia*-infected and/or uninfected mosquitos. Maternal transmission of *Wolbachia* effects. **(A)** When *Wolbachia* uninfected female and male mosquitoes mate, they will give a viable egg that will continue the next generation and disease transmission. To tackle it, intervention, IIT, and/or SIT are needed. Similarly, **(B)** when infected females and males mate, they produce infected viable eggs. However, the development of the offspring continues the phenotype, adult behaviors may change, the life span is short, and the population declines. As a result, no more diseases frequently occur, but intervention like SIT is still needed. **(C)** When infected females mate with uninfected males, they produce infected viable eggs that can grow but are less susceptible to developing pathogens and transmitting diseases. The adult life span may be short and may not effectively bite humans and other hosts. Nevertheless, control intervention such as SIT is needed. **(D)** When an uninfected female mating with an infected male, no more viable egg is produced or early embryonic death occurs in the new generation. As a result, no more intervention is needed. Key: W+: *Wolbachia* infected, W−: *Wolbachia* uninfected, ×: Mates.

*Wolbachia* infection on *Aedes* is becoming an increasingly popular alternative candidate strategy for controlling vector-borne disease transmission (Brelsfoard and Dobson, 2011). According to research findings, infected females can successfully mate with infected and uninfected males and give live *Wolbachia*-positive offspring (Sinkins, 2004; O’Neill, 2018). On the other hand, when uninfected females mate with infected males, they produce non-viable eggs (Figure 3) (Charlat et al., 2001; Poinsot et al., 2003; Werren et al., 2008; Brelsfoard and Dobson, 2011; Beebe et al., 2021). As a result of male sperm infection, haploid cells do not effectively fuse with uninfected eggs, causing the failure of embryonic development or early embryonic death (Caragata et al., 2021). Other research findings pointed out that *Wolbachia*-infected male nutrition can be linked to reduced fertility and fecundity in mates (Islam and Dobson, 2006; Beebe et al., 2021). This disrupts the normal development of the zygote produced by infected males and uninfected females mating (Serbus et al., 2008).

The study conducted in Crevalcore, Italy by Puggioli et al. (2016) focused on using a specific *Ae. albopictus* line that was genetically modified to produce sterile males generated by introducing wPip in the ARwP line. The finding showed bidirectional reproductive barriers between infected and uninfected mosquitoes, meaning that when infected males mate with uninfected females or vice versa, the eggs produced fail to develop or hatch, thus leading to a reduction in the overall mosquito population (Puggioli et al., 2016). Similarly, the study conducted by Moretti et al. (2018) in Italy focused on a genetically manipulated line of *Ae. albopictus* mosquitoes using ARwP-M reduced *Ae. albopictus* populations. This suggested the introduction of a combined ARwP-M line, which carries wPip and wMel-induced sterility and virus protection to fight *Ae. Albopictus-borne* viruses. This could be a potential strategy for controlling *Ae. albopictus* populations and reducing the transmission of chikungunya and dengue viruses (Moretti et al., 2018).

Based on this, to reduce the *Ae. albopictus* population in the field, Zheng et al. (2019) used Incompatible Insect Technology (IIT), which utilizes sterilization with the maternally inherited endosymbiont *Wolbachia*, but the accidental release of females infected with the same strain of *Wolbachia* as the released males could comprise its effectiveness (Zheng et al., 2019). In this scenario, a study conducted in Nanyang, Singapore by Ong et al. (2022) reported that an IIT combined with a sterile insect technique (IIT-SIT) releasing X-ray irradiated *Wolbachia*-infected male mosquitoes resulted in a 98% reduction in *Ae. aegypti* populations also showed an 88% reduction in the incidence of dengue fever (Ong et al., 2022).

Likewise, an on-field trial performed in South Miami, United States, by Mains et al. (2019) showed the release of many infected *Ae. aegypti* males significantly reduced the egg-hatching rate in areas populated by infected males, consistent with the CI expectations. Similarly, the number of *Ae. aegypti* were significantly reduced in areas where infected males were getting infected compared to untreated areas, reducing the Zika virus burden (Mains et al., 2019). Moreover, the release of sterile or incompatible males resulted in the suppression of both wild-type and wMel-infected A*e.aegypti* populations, confirming the utility of bidirectional incompatibility in the field (Beebe et al., 2021) as demonstrated in northern Queensland, Australia by Beebe et al. (2021).

The main limitation of the IIT is releasing infected male mosquitos maternally inherited. To overcome this constraint, a combination strategy between IIT techniques and the Sterile Insect Technique (SIT) was also tested, whereby overwhelming numbers of sterile insects are released into the wild (Zheng et al., 2019; Villegas-Chim et al., 2022). SIT is a control method applied against agricultural pests as well as human disease vector populations, by providing the release of sterile or incompatible males (Werren et al., 2008). According to Iturbe-Ormaetxe et al. (2011), *Wolbachia* transinfection experiments are more successful when the donor and recipient organisms are closely related.

Likewise, Moreira et al. (2009a,b) and Bian et al. (2010) demonstrated that bacterial infection can occur in different body parts of *Ae.aegypti* like the midgut, fat body, brain, and salivary gland, with a high prevalence in the reproductive tissues, both ovaries and testicles (Bi and Wang, 2020). Another study conducted in Mexico by Mejia et al. (2022) found relatively greater *Wolbachia* densities in reproductive tissues than those in the somatic tissues (Mejia et al., 2022). This implies that reproductive parts infection can inhibit the vector fecundity and fertility.

Beyond the reproductive system, the brain also is a target for *Wolbachia* infection, affecting oviposition and host-seeking behavior. However, this condition does not alter the attraction of mosquitoes to the human odor (Wiwatanaratanabutr et al., 2010; Turley et al., 2014) but rather, the blood-feeding ability by affecting the proboscis’s anatomy (Turley et al., 2009; Moreira et al., 2009a; Bian et al., 2010).

A study by Caragata et al. (2016a) demonstrated that *Wolbachia* infection can induce diet-nutritional stress in *Ae. aegypti* reducing vector susceptibility versus Dengue virus and the avian malarial parasite *Plasmodium gallinaceum*. Similarly, Geoghegan et al. (2017) found that infection alters lipid/cholesterol metabolism including differential cholesterol and lipid profiles (Geoghegan et al., 2017). These findings suggest a possible competition for nutrients between *Wolbachia* and other pathogens inhibiting replication and shortening vector life span.

According to De Oliveira et al. (2017), *Ae. albopictus* demonstrated greater competitive ability in a variety of laboratory settings. Its larvae outperformed *Ae. aegypti* (both infected and uninfected groups) in terms of development and performance index survival rate. *Wolbachia* boosted the larval survival rate of *Ae. aegypti.* This finding indicated that larval density greatly impacts the competition for nutrients in infected vectors (De Oliveira et al., 2017).

According to a study conducted by Islam and Dobson (2006) on the impact of *Wolbachia* on *Ae. albopictus,* uninfected larvae, had the best survival rate, partly because males infected with wAlbB or wAlbA had lower survival rates. Dutra et al. (2016), recorded similar results at Penn State Brazil and found that wMel infection of *Ae. aegypti* caused faster larval growth in males and females at greater densities but did not affect females living in less crowded settings (Dutra et al., 2016). While wMelPop infection of *Ae. aegypti* exhibited highly inhibitory effects of larval food level, the effect of strain alone was not significant, according to a different study conducted in Queensland, Australia by Kho et al. (2016). These differences may be due to bacterial density and host susceptibility. Thus a higher density causes more pronounced effects. For instance, *W. pipientis* strain wMelPop is known for shortening life spans when inserted into the main dengue vector *Ae. aegypti* (Thomas et al., 2011; Yeap et al., 2014) but not in *Ae. albopictus* (Mousson et al., 2010), as demonstrated by Wiwatanaratanabutr et al. (2010).

According to Ross et al. (2017) wMel and wMelPop-CLA infections of *Ae. aegypti* could not be transmitted to the next generation when mosquitoes were exposed to 26–37°C across all life stages. In contrast, under the same temperature range, an increase in infection density allowed the infection to be inherited from mother to offspring (Ross et al., 2017).

Besides vertical transmission, innate immune priming is also strain and density-dependent. Indeed, epithelial cells that are also involved in regulating innate immune responses to bacteria and parasites produce a significant number of reactive oxygen species and antimicrobial peptides (Ryu et al., 2010; Pircalabioru et al., 2016). In *Ae. aegypti,* an increased level of reactive oxygen compounds suppresses the replication of West Nile virus (Hussain et al., 2013), Dengue virus (Bian et al., 2010; Frentiu et al., 2014), Chikungunya virus (Aliota et al., 2016a), and Zika virus (Aliota et al., 2016b). Furthermore, it confers resistance to various ribonucleic acid (RNA) viruses and virus-induced death in flies, but it reduces adult vectorial capacity (Mohanty et al., 2016). Replication of West Nile virus is significantly reduced in the presence of *Wolbachia* by the alteration of GATA4 expression which inhibits virus assembly (Hussain et al., 2013). Those imply that *Wolbachia* infection has evolutionary, biological, and developmental impacts on mosquito vectors.

On the other hand, Quek et al. (2022) found that in the absence of *Wolbachia*, microfilariae quickly lose their capacity to develop in the mosquito vector because of their inability to break out of their shells and get through the gut wall. They also showed that the enzyme chitinase, essential for microfilariae to leave their shells, was low in *Wolbachia*-depleted microfilariae, preventing them from leaving their shells. When chitinase was added to *Wolbachia*-depleted microfilariae in a lab, they could break out of their shells just as well as the ones that were not treated. So, it looks like *Wolbachia* has a big role in the transmission of filariasis and suggests that anti-*Wolbachia* treatment mediates a more accelerated impact on the elimination of lymphatic filariasis (Quek et al., 2022).

### 2.2. *Wolbachia* infection in *Anopheles* mosquitoes

Among all retrieved articles in this review, 13 were on *Wolbachia* infection of *Anopheles* species (Supplementary Figure S1 and Supplementary Table S1). There are more than 460 recognized species in the genus *Anopheles. An. gambiae* and *An. funestus* are the most significant global malaria vectors (Sinka et al., 2012; Wiebe et al., 2017). Currently, *An.stepheni* is going to be the main concern for malaria transmission in Africa. The genus *Anopheles* is most well-known for conveying malaria but also transmits other diseases like filarial worms (Coetzee, 2020; Kientega et al., 2022).

The first recorded on-field infection of *Wolbachia* in *Anopheles* species was reported in Burkina Faso by Baldini et al. (2014) using 16S rRNA gene analysis from *An. gambiae* reproductive tissue. This study also isolated a new strain of *Wolbachia*, namely wAnga. Similarly, other research conducted in Senegal reported the first *Wolbachia* on-field infection in another species, namely *An.funestus*, using the 16S rRNA gene and isolating new strains called wAnfu-A and wAnfu-B (Niang et al., 2018). In 2022 Waymire et al. detected *Wolbachia* haplotypes in wild *Anopheles stephensi* in eastern Ethiopia (Waymire et al., 2022). Despite this evidence, according to a phylogenies screening conducted in 2019 by Chrostek and Gerth on *Wolbachia* 16S rRNA presence in *An. gambiae* there is no congruence between host and symbiont phylogenies (Chrostek and Gerth, 2019).

#### 2.2.1. *Wolbachia* infection and parasite development *Anopheles* species

Once the *Anopheles* infection is established, the inherent mechanism is similar to the *Aedes* and *Culex* species, as shown in Figure 2 (Hughes et al., 2011). However, the role of *Wolbachia* in inhibiting the malaria parasites in *Anopheles* mosquitoes is still not well-known (Straub et al., 2020), *In vitro* trans-infection of *An. gambiae* with wMelPop and wAlbB strains performed by Hughes et al. (2011) demonstrated the bacteria distributed throughout the fat body, head, sensory organs, and other tissues.

On the other hand, a study conducted in Pennsylvania, United States, by Hughes et al. (2012) found that the wAlbB strain significantly increases *P. berghei* oocyst levels in the infected *An. gambiae* midgut while wMelPop modestly suppresses oocyst levels. Another study from East Lansing, United States, by Joshi et al. (2014), reported that wAlbB infections of *An. stephensi* had reduced female fecundity and caused a minor decrease in male mating competitiveness. Later, Joshi et al. (2017) revealed wAlbB infection in *An. stephensi* led to a reduction in parasite numbers of up to 92% at the sporozoite stage and more than half at the oocyst stage. This finding implies that wAlbB strain infections can reduce the parasite density depending on the *Plasmodium* species and vector population. This evidence is in agreement with what was reported by Baldini et al. (2018) on natural *Wolbachia* infection in the malaria mosquito *Anopheles arab*iensis in Tanzania.

Another study conducted in Dangassa, Mali, by Gomes et al. (2017) found the *Wolbachia* infection in the field-collected *An. coluzzii* was positive for wAnga and revealed a significantly lower prevalence and intensity of *P. falciparum* sporozoite. Similarly, in Bobo-Dioulasso, Burkina Faso, Shaw et al. (2016) revealed that *Wolbachia* infections in natural *Anopheles* populations affect egg laying and negatively correlate with *Plasmodium* development. Finally, in 2020 Wong et al. reported that *Wolbachia* infection in *An. gambiae* is able to reduce the mosquito life span and provide resistance to pathogen infection (Wong et al., 2020).

### 2.3. *Wolbachia* infection in *Culex* species

Among all retrieved articles in this review, 11 have as subjects the epidemiology and infection of *Culex* species (Supplementary Table S1). The genus *Culex* has several species; however, *Cx. pipiens* and *Cx. quinquefasciatus,* reviewed in the selected studies, are vectors for various human diseases, such as arbovirus diseases like the *West Nile* virus, Japanese encephalitis, and filariasis (Harbach, 1985; Omar, 1996; Paramasivan et al., 2003).

The first *Wolbachia* infection in mosquitoes was reported from *Cx. pipiens* reproductive tissues by Hertig and Wolbach (1924). Later on, different studies showed that the prevalence of *Wolbachia* in this species ranges from 65% to 100% in field-collected females and nearly 100% in males (Karami et al., 2016; Bergman and Hesson, 2021).

#### 2.3.1. *Wolbachia* infection and its effect on the *Culex* species

The mode of infection and its effect on *Culex* physiology is similar to *Aedes*. However, Hague et al. (2020) demonstrated that the infected host raises temperature preference. In contrast to the uninfected, most hosts infected with *Wolbachia* supergroup A prefer cooler temperatures than uninfected ones, On the other hand, supergroup B infected hosts prefer warmer temperatures (Hague et al., 2020). These findings suggest that *Wolbachia* infection-inducing host behavior’s alterations facilitate bacterial replication and disease spread (Moreira et al., 2009b; Glaser and Meola, 2010; Caragata et al., 2016b). Interestingly similar evidence has not been reported for *Aedes* and *Anopheles.*

According to Atyame et al. (2011) a considerable amount of *Wolbachia* diversity can be generated within a single host species in a short time, and playing a key role in their evolution. Furthermore, a recent study by Zhang et al. (2020) clarified the immune system’s role in *Cx. pipiens* infection, according to the author the competition for scarce nutrients may not be the primary cause of *Wolbachia*-mediated pathogen suppression, as evidenced by the fact that the presence of *Wolbachia per se* does not always alter pathogen infections. Instead, it is brought on by host immunological reactions (Zhang et al., 2020).

*In vitro* insecticide susceptibility studies by Berticat et al. (2002) and Duron et al. (2006) showed that the symbiotic maternally inherited *Wolbachia* affected *Cx. quinquefasciatus* and *Cx. pipiens* insecticide resistance depending on infection density and the type of insecticide used (Berticat et al., 2002; Duron et al., 2006). Therefore, a medium-density infection synergises with deltamethrin and other organophosphates, but not with Dichloro-diphenyl-trichloroethane (DDT) (Berticat et al., 2002; Duron et al., 2006; Shemshadian et al., 2021). Likewise, Echaubard et al. (2010) also reported that a medium-density infection in *Cx. pipiens* made the vector insecticide-susceptible whereas with a higher density infection caused insecticide-resistance. These results may partially explain the presence of high-density infections in pesticide-resistant mosquitoes in the field (Echaubard et al., 2010).

Similarly, *in vitro* infection with wPipSJ made *Cx. quinquefasciatus* less susceptible to entomopathogenic bacteria as demonstrated by Díaz-Nieto et al. (2021). These findings agree with on-field records, where *Cx. quinquefasciatus* infected by wPipSJ are more resistant to *Bacillus wiedmannii* var. *thuringiensis*, and *B. thuringiensis* subsp. israelensis, and *Lysinibacillus sphaericus* bacterial infections (Díaz-Nieto et al., 2021).

## 3. Discussion

Integrated Mosquito Management (IMM) strategies are currently the best option for reducing mosquito populations (CDC, 2020). This implementation is based on understanding mosquito biology, ecology, and mosquito pathogen interaction. Indeed, IMM programs employ several strategies, together with insecticides, such as larval breeding source reduction through community participation and biological control techniques like predatory fish and symbiotic bacteria (Dodson et al., 2017; CDC, 2020).

Currently, scientific evidence has underscored the appropriate use of the symbiotic bacteria *W. pipientis* as a new weapon in the fight against mosquitoes as vector-borne diseases. Compared to insecticide-based methods, it has the advantage of potentially being more cost-effective and environmentally friendly (Iturbe-Ormaetxe et al., 2011). In addition, *Wolbachia* infection density was positively correlated with insecticides, making this management strategy even more attractive (Berticat et al., 2002; Duron et al., 2006; Shemshadian et al., 2021). These suggest that reducing vector population and other pathogen replication in the host also increases the vectors’ susceptibility to different insecticides.

When considering *Wolbachia* infection as a pathogen for inhibition and population reduction, factors such as strain, density, distribution, and infection frequency must be considered (Bian et al., 2010). The mechanism of *Wolbachia* infection to protect the host from pathogens is immune priming, in which symbiotic infection upregulates basal immune responses and primes insect defenses against subsequent pathogen infections (Ye et al., 2013). However, Hughes et al. (2012) reported that the wAlbB strain significantly increases *P. berghei* oocyst levels in the infected *An. gambiae*. These various effects imply that *Wolbachia* strains differ in their interactions with the host and/or pathogen, and these variations may be used to elucidate the molecular processes that prevent pathogen development in mosquitoes.

In addition to strain and density, the distribution of bacterial infections within the mosquito’s body also significantly impacts mosquito population decline. Infection of the reproductive tract causes host reproductive failure due to CI (Li et al., 2023). Reproductively infected mosquitoes cannot produce viable offspring or transmit the bacteria to their offspring (Sinkins, 2004; O’Neill, 2018). On this basis, releasing *Wolbachia*-infected male mosquitoes into the field decreased the fecundity and the fertility of wild mosquito populations. *Wolbachia* Incompatible Insect Technology (IIT) performing this strategy has proven to be a promising method for eliminating invasive mosquito populations such as *Ae. aegypti* and *Ae. albopictus* and reducing the incidence of vector-borne diseases such as dengue, chikungunya, and Zika (Pagendam et al., 2020).

To enhance the effect of population reduction of mosquitoes in the human community, IIT can be combined with radiation-based SIT, which is rearing, sterilization, and release of large numbers of male mosquitoes to mate with fertile wild females, thereby reducing offspring production from the target population (Zheng et al., 2019; Chen et al., 2023). This further reinforces the dependence on strain type and density in infection vertical transmission.

Even though the release of *Wolbachia*-carrying mosquitoes into communities is not immediately stopping the epidemic, it leads to mosquito population declines over several months (Iturbe-Ormaetxe et al., 2011; Liew et al., 2021). These imply that *Wolbachia* influences the transmission effect when mosquitoes are exposed for an extended time to obtain the capacity of *Wolbachia* strains to infiltrate the uninfected mosquito population in the community.

Besides, before and during the implementation of releases of *Wolbachia*-infected mosquitoes for mosquito population suppression or replacement. It is important to keep engaging with the community and educating them to increase their understanding of this method, including clear and specific health risk assessment information (Sánchez-González et al., 2021). In addition to maintaining community support, programs should evaluate and monitor to determine how well they reduce the mosquito population (Sánchez-González et al., 2021; Villegas-Chim et al., 2022). Household perception surveys in different areas of Singapore provided a good understanding of public acceptance and sentiments toward using *Wolbachia-Aedes* technology (Liew et al., 2021). In addition, Texas and California in the United States, Thailand, Mexico, and Australia have released *Wolbachia*-infected mosquitoes and reported a significant drop in *Ae. aegypti* mosquitoes to control dengue, chikungunya, and Zika also gaining acceptance in the community (Wiwatanaratanabutr et al., 2010; Torres et al., 2020; Villegas-Chim et al., 2022).

The main difficulty with using *Wolbachia* for controlling vectors in the community is that the main vectors, like *Ae.aegypti* and *Anopheles* species, are not usually naturally infected. Trans-infection in the laboratory is necessary to ensure the bacterium is stably transmitted in these vector populations. In addition, culturing obligate intracellular bacteria is a challenge. Insect cells support *Wolbachia* growth, but culturing is long and difficult to manipulate cells. Modified Eagle’s Minimum Essential Medium, Schneider’s Insect Medium, Mitsuhashi-Maramorosch Insect Medium, and their one-to-one combinations are tested and effective for *Wolbachia* culture (Angeloni, 2021). Moreover, experts must transfer *Wolbachia’s* strain into a new host once it grows in the cell culture.

## 4. Conclusion

Different *Wolbachia* species and strains have been isolated at different times. These different *Wolbachia* species and strains commonly infect and affect mosquito species differently. Once *Wolbachia*-infected mosquitoes release, they may reduce or prevent disease transmission through two mechanisms: (1) by reducing mosquito population density and/or survival rate; (2) by reducing the ability of mosquitoes to transmit diseases and/or pathogen replication or development. It causes the hosts’ CI, phenotypic changes, and nutritional competition with other pathogens. These triggers reduce adult survivorship, inhibit mosquito reproduction, and prevent pathogen replication or development. *Wolbachia* infection from mosquitoes also sensitizes status to insecticides. Accordingly, *Wolbachia* can be used for biological control of mosquito-borne diseases, a public health problem in the tropical and sub-tropical world and some developed countries. *Wolbachia* reduces infection and transmission of diseases such as malaria, filariasis, dengue, chikungunya, yellow fever, zika, and West Nile fever.

## Author contributions

AM: Conceptualization, Formal analysis, Writing – original draft, Writing – review & editing, Investigation, Validation, Visualization. GPP: Validation, Visualization, Writing – review & editing, Investigation, Supervision. DY: Validation, Writing – review & editing, Supervision. AS: Supervision, Validation, Writing – review & editing. IM: Writing – review & editing, Validation. DN: Validation, Writing – review & editing. RM: Supervision, Validation, Writing – review & editing. GA: Supervision, Validation, Writing – review & editing, Conceptualization, Data curation.

## Abbreviations

AMP: Antimicrobial peptides
CHIKV: Chikungunya virus
CI: Cytoplasmic incompatibility
DDT: Dichlorodiphenyltrichloroethane
DENV: Dengue Virus
IIT: Incompatible insect technique
IMM: Integrated mosquito management
NO: Nitric oxide
PCR: Polymerized chain reaction
RNA: Ribonucleic acid
ROS: Reactive oxygen species
SIT: Sterile insect technique
