# Supplementary material for: Symbiotic Wolbachia in mosquitoes and its role in reducing the transmission of mosquito-borne diseases: updates and prospects
Source: Front Microbiol. 2023 Oct 13;14:1267832. doi: 10.3389/fmicb.2023.1267832 (PMC10612335; doi:10.3389/fmicb.2023.1267832)
Supplement: Supplementary file 1 [file Data_Sheet_1.docx]

**Supplementary Material**

**Supplementary Figure 1.** Flowchart of study selection for review Symbiotic *Wolbachia* in mosquitoes and its role in reducing the transmission of mosquito-borne diseases: updates and prospects


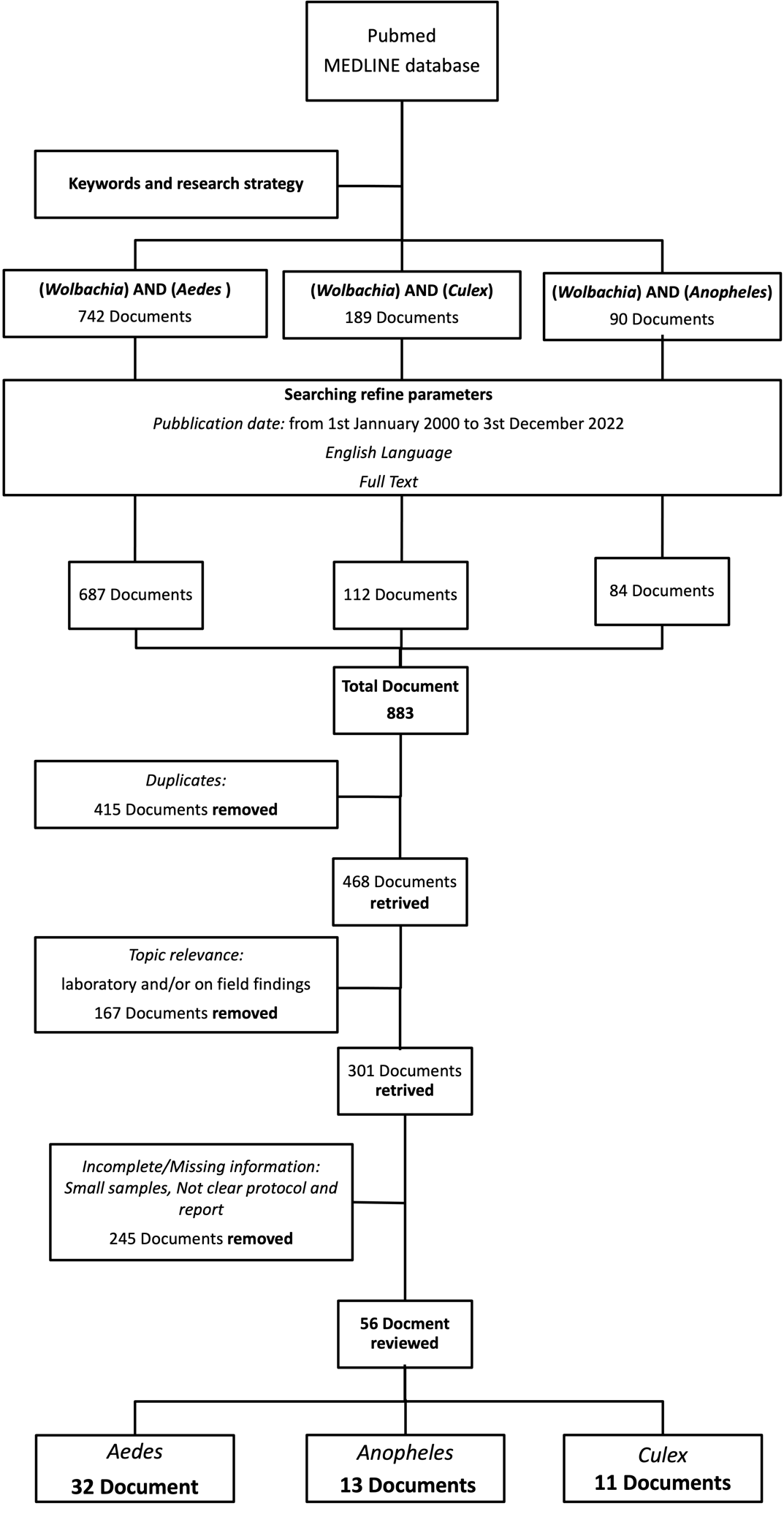


**Supplementary Table 1.** Field infected and trans-infection of mosquito by Wolbachia and its prevention effect of vector-borne diseases.

| Topic | *Wolbachia* strain | Vector/s species | | Research strategy & methodology | Main findings |
| --- | --- | --- | --- | --- | --- |
| *Aedes* spp. | | | | | |
| Epidemiology | wAlb and Pip | *Ae. albopictus* | | PCR based approach | Maternal transmission is nearly 100% [1] |
|  | *Wolbachia* | *Ae. aegypti* | | Trans-infection technique | Inherited microorganisms [2] |
|  | Wsp | *Ae. albopictus* | | On field-collected PCR-based approach | Infections prevalence 86.7% [3] |
| Infection | Wsp | *Aedes* and *Culex* | | PCR-based approach | Predominately found in the reproductive tissues but infection rates varied according to host species [4] |
|  | wAlbA+wAlbB | *Ae. albopictus* | | PCR-based and antibody-based approaches | Distributed in host somatic and germ-line tissues [5] |
|  | Wsp | *Ae. albopictus* | | On-field infection and PCR-based approach | Modify vector populations and deliver pathogen-blocking genes [6] |
|  | Wsp | *Ae. albopictus* | | PCR-based approach | No differences in oviposition responses and strain-specific differences in mating, and host-seeking behavior [7] |
|  | wAlbA &  wAlbB | *Ae. albopictus* | | PCR-based approach | Effect on immature survivorship, developmental rate, adult size, larval nutrition, and CI level causing reduced larval survival and food computation [8] |
|  | wPip | *Ae. albopictus* | | Genes-editing approach | Bidirectional reproductive barriers between infected and uninfected mosquitoes [9] |
|  | wPip , wMel, wAlbA &  wAlbB | *Ae. albopictus* | | Induced sterility and virus protection | Reducing transmission of CHIKV and DENV [10] |
|  | Mel, AlbA and Pip | *Ae. albopictus,*  *Cx.quinquefasciatus* | | Field data screened by PCR-based approach s | Reduce viral and other bacterial replication [11] |
|  | wMelPop | *Ae. aegypti* | | PCR-based and antibody-based approaches | Predominately found in the fat body, Malphgian tubules, ovaries, and Salivary glands, inducing phenotype change and adult lifespan reduction [12] |
|  | wMelPop | *Ae. aegypti* | | PCR-based approaches | Anatomical changes, unable to successfully insert stylet into human skin, reducing feeding success, Infection and lifespan [13] |
|  | wMelPop | *Ae. aegypti* | | Blood-feeding trials in response to humans | Anatomical changes, "bendy" proboscis, reducing feeding success, Infection and lifespan [14] |
|  | wMel | *Ae. aegypti* | | In field challenge and PCR-based approach | Infected and uninfected distributed in the same body tissues, wMel reduce viral infection [15] |
|  | *wMelPop-CLA* | *Ae. aegypti* | | Morphometric assessments | Infected mosquitoes have low infection fitness in the field [16] |
|  | wMelPop | *Ae. aegypti* | | Microarray and PCR-based approach | Vector was less susceptible to filariasis infection by immune responses up regulation and sorter lifespan [17] |
|  | wMel | *Ae. aegypti* | | Laboratory larval competition | Density and sex dependent effect, males development inhibition [18] |
|  | wMel | *Ae. aegypti* | | Effects of infected larval nutrition | Both nutrition level and infection showed interference in larval development and diet one also showed Larval development is impaired in infected mosquitoes but not strain [19] |
|  | wMel | *Ae. aegypti* | | Odor cues response in the field | Reduction of adult lifespan and blood feeding success not alteration of odor attraction [20] |
|  | wMel | *Ae. aegypti* | | *Wolbachia*-infected mosquitoes release into the field | Reduction of mosquito populations and virus-amplification in the female mosquito [21] |
|  | wAlbB-wsp | *Ae. aegypt* | | PCR-based approaches | Density-dependent increased CI basal immunity and longevity, reduced lifespan [22] |
|  | wMelBr | *Ae. albopictus*  *Ae. aegypti* | | Wild and trans-infected mosquitoes interspecific competition | *Larvae* had superior survivorship, faster development rate and a higher performance index in *Ae albupictus* than *Ae. aegypti* both infected and uninfected groups [23] |
|  | wMel | *Ae. aegypti* | | PCR-based approach | Resources competition induced gene expression, metabolic homeostasis, and physiological processes causing ROS and NOs expression [24] |
|  | wMelPop | *Ae. aegypti* | | PCR-based approach | Infection alters lipid/cholesterol metabolism including differential cholesterol and lipid profiles [25] |
|  | wMelPop-CLA | *Ae. aegypti* | | PCR-based approach | Strain-dependent GATA4 expression inhibiting virus assembly [26] |
|  | Different strain | *Ae. aegypti* | | *Wolbachia* usage and surveillance | High support from the community [27] |
|  | *wMel* | *Ae. aegypti* | | PCR-based approach | Reduced vector competence for ZIKV [28] |
|  | wMelPop | *Ae. aegypti* | | PCR-based approach | Long period infection reduces adult life span and DENV transmission [29] |
|  | wMelPop | *Ae. aegypti* | | On-Field blood meal, PCR based and antibody-based approaches | Innate immune system priming and limited cellular resources are required for pathogen replication [30] |
|  | *wMel* | *Ae. aegypti* | | Released *Wolbachia*-infected mosquito | Reduced vector competence for CHIKV, extremely high viral titers detected in the blood meal but no infectious virus in saliva [31] |
|  | wBm | *Ae. aegypti.* | | PCR-based and antibody-based approaches | *Wolbachia* depletion blocks transmission of lymphatic filariasis preventing chitinase-dependent parasite escheatment, causes depletion of *Wolbachia* , leads to a block of MF development to stage 3 [32] |
| *Anopheles* spp. | | | | | |
| Epidemiology | coxA haplotypes | | *An. stephensi* | PCR-based approaches | 10.9% of *An. stephensi* screened positive from *An. stephensi* with multiple haplotypes and supergroups A and B [33] |
|  | Wsp | | *An. gambiae* | PCR-based approaches | High *Wolbachia* 16S rRNA sequence diversity, low abundance, and no congruence between host and symbiont phylogenies *Wolbachia* sequences were detected [34] |
|  | wAnfu-A and wAnfu-B | | *An. fenstusts* | PCR-based approaches | New strains were isolated, the discovery of natural infection [35] |
|  | Wanga | | *An. gambiae* | PCR-based approaches | Naturally harbor these bacteria and Novel *Wolbachia* strain, isolated [36] |
| Infection | *wAlbB* | | *An. stephensi* | PCR-based approaches | Reduced female fecundity and caused a minor decrease in male mating competitiveness [37] |
|  | *wAlbB* | | *An. stephensi* | PCR-based approaches | Reduction in parasite numbers of up to 92% at the sporozoite stage and more than half at the oocyst stage [38] |
|  | *Wsp* | | *An. gambiae* | PCR-based approaches | natural infection, reducing the life span of vectors and providing resistance to pathogen infection [39] |
|  | wAlbB,  wMelPop | | *An. gambiae* | PCR-based approaches | Differences in interference of pathogen development in mosquitoes. The wAlbB strain significantly increases *P. berghei* oocyst levels in the mosquito midgut while wMelPop modestly suppresses oocyst levels [40] |
|  | *wMelPop and wAlbB* | | *An.*  *gambiae* | PCR-based and antibody-based approaches | wMelPop disseminates widely in the fat body, head, sensory organs and other tissues but is absent from the midgut and ovaries, significantly inhibiting *P.falciparum* oocyst levels in the mosquito midgut [41] |
|  | Wanga | | *An. gambiae , An. coluzzii* | PCR-based approaches | Relatively higher prevalence and intensity of infection-detected significantly reduced the prevalence and intensity of sporozoite infection [42] |
|  | WAnga-M | | *An.gambiae* and *An. coluzzii* | PCR-based approaches | wAnga influenced mosquito egg-laying behaviour ,phenotypic change of the vector, inhibition to *P. falciparum* development [43] |
|  | WAnga | | *An. coluzzii,* | PCR based and antibody-based approaches | negative correlation between the presence of *Plasmodium* parasites and *Wolbachia* infection, wAnga does not induce CI but affects oviposition to reduce malaria prevalence [44] |
|  | Wanga | | *An. arabiensis & An. funestus,* | PCR-based approaches | confirmation of natural *Wolbachia* in malaria vectors but low prevalence and density within species, limits the malaria parasite *P. falciparum* infections [45] |
| *Culex* spp. | | | | | |
| Epidemiology | Wsp | | *Cx pipiens* | PCR-based approaches | Prevalence 87.3% wild-caught mosquitoes, rate of infection females 61.5% to 100% while males 80% to 100%. horizontal transfer between unrelated host organisms, results in a high proportion of infection prevalence and rate [46] |
|  | Wsp | | *Culex pipiens and Culex torrentium* | PCR-based approaches | The prevalence of *Wolbachia* in *Cx. pipiens* was 97% (95% CI 94.8–97.6%), while only 0.7% (95% CI 0.19–2.45%) in *Cx. torrentium* [47] |
| Infection | Group A and B | | *Different strains* | PCR-based approaches | Wolbachia supergroup A prefer cooler temperatures than uninfected ones, On the other hand, supergroup B infected-hosts prefer warmer temperatures [48] |
|  | Wolbachia spp. | | *Cx. quinquefasciatus* | PCR-based approaches | *Wolbachia* reduces vector competence in *Cx. quinquefasciatus*, and potentially in other Wolbachia-infected mosquito vectors [49] |
|  | Different strains | | *Cx. pipiens* | PCR-based approaches | A new host-*Wolbachia* symbioses had a higher survival rate than in old host-*Wolbachia* symbioses. Induction of host innate immune responses [50] |
|  | Wpip | | *Cx. pipiens* | PCR-based approaches | A considerable amount of *Wolbachia* diversity can be generated within a single host species in a short time, and playing a key role in their evolution [51] |
|  | *Different strain* | | *Cx pipiens* | Insecticide resistance stages | High infection densities do not increase CI or maternal transmission efficiency relative to low infection densities and increase insecticide resistance. Increased density could contribute to the fitness cost of resistance [52] |
|  | Different strain | | *Cx. pipiens* | PCR-based approaches | Insecticide-resistant strains, sharing the same cytoplasmic and nuclear background has an insecticide-susceptible strain, the higher *Wolbachia* density, correlated to insecticide resistance, and increase the physiological costs of insecticide resistance [53] |
|  | Different strain | | *Cx pipiens.* | PCR-based approaches | Strains harboring the resistance gene had more density than a susceptible strain with the same genetic background, the resistant mosquitoes had significantly higher infection rates than the susceptible ones [54] |
|  | Different strain | | *Cx quinquefasciatus* | PCR-based approaches | No correlation between susceptibility of two lines in response to DDT while they represented a significant correlation for deltamethrin, infection increased the susceptibility to deltamethrin but had a neutral effect on DDT susceptibility [55] |
|  | WpipSJ | | *Cx. Quinquefasciatus* | PCR-based approaches | Native wPipSJ infection is more resistant to the pathogenic action of the three mosquitocidal bacterial strains, naturally infected with wPipSJ is less susceptible, while susceptible to *B. thuringiensis* subsp. israelensis than to the other [56] |

coxA: cytochrome c oxidase subunit, CI: cytoplasmic incompatibility; CHIKV: Chikungunya virus; DENV: dengue virus, Wsp: *Wolbachia* surface antigen, MF: Microfilaria.

**Table references**

1. Kittayapong P, Baimai V, O'Neill SL. Field prevalence of Wolbachia in the mosquito vector Aedes albopictus. American Journal of Tropical Medicine and Hygiene. 2002;66(1):108-11.

2. O’Neill SL. The use of Wolbachia by the World Mosquito Program to interrupt transmission of Aedes aegypti transmitted viruses. Dengue and Zika: control and antiviral treatment strategies. 2018:355-60.

3. Li Y, Sun Y, Zou J, Zhong D, Liu R, Zhu C, et al. Characterizing the Wolbachia infection in field-collected Culicidae mosquitoes from Hainan Province, China. Parasites & vectors. 2023;16(1):1-12.

4. Ding H, Yeo H, Puniamoorthy N. Wolbachia infection in wild mosquitoes (Diptera: Culicidae): implications for transmission modes and host-endosymbiont associations in Singapore. Parasites & Vectors. 2020;13(1):1-16.

5. Dobson SL, Bourtzis K, Braig HR, Jones BF, Zhou W, Rousset F, et al. Wolbachia infections are distributed throughout insect somatic and germ line tissues. Insect biochemistry and molecular biology. 1999;29(2):153-60.

6. Kittayapong P, Baisley KJ, Baimai V, O’Neill SL. Distribution and diversity of Wolbachia infections in Southeast Asian mosquitoes (Diptera: Culicidae). Journal of medical entomology. 2000;37(3):340-5.

7. Wiwatanaratanabutr I, Allan S, Linthicum K, Kittayapong P. Strain-specific differences in mating, oviposition, and host-seeking behavior between Wolbachia-infected and uninfected Aedes albopictus. Journal of the American Mosquito Control Association. 2010;26(3):265-73.

8. Islam MS, Dobson SL. Wolbachia effects on Aedes albopictus (Diptera: Culicidae) immature survivorship and development. Journal of medical entomology. 2006;43(4):689-95.

9. Puggioli A, Calvitti M, Moretti R, Bellini R. wPip Wolbachia contribution to Aedes albopictus SIT performance: advantages under intensive rearing. Acta Tropica. 2016;164:473-81.

10. Moretti R, Yen P-S, Houé V, Lampazzi E, Desiderio A, Failloux A-B, et al. Combining Wolbachia-induced sterility and virus protection to fight Aedes albopictus-borne viruses. PLoS neglected tropical diseases. 2018;12(7):e0006626.

11. Zheng X, Zhang D, Li Y, Yang C, Wu Y, Liang X, et al. Incompatible and sterile insect techniques combined eliminate mosquitoes. Nature. 2019;572(7767):56-61.

12. Walker T, Johnson P, Moreira L, Iturbe-Ormaetxe I, Frentiu F, McMeniman C, et al. The w Mel Wolbachia strain blocks dengue and invades caged Aedes aegypti populations. Nature. 2011;476(7361):450-3.

13. Moreira LA, Iturbe-Ormaetxe I, Jeffery JA, Lu G, Pyke AT, Hedges LM, et al. A Wolbachia symbiont in Aedes aegypti limits infection with dengue, Chikungunya, and Plasmodium. Cell. 2009;139(7):1268-78.

14. Turley AP, Moreira LA, O'Neill SL, McGraw EA. Wolbachia infection reduces blood-feeding success in the dengue fever mosquito, Aedes aegypti. PLoS Neglected Tropical Diseases. 2009;3(9):e516.

15. Frentiu FD, Zakir T, Walker T, Popovici J, Pyke AT, van den Hurk A, et al. Limited dengue virus replication in field-collected Aedes aegypti mosquitoes infected with Wolbachia. PLoS neglected tropical diseases. 2014;8(2):e2688.

16. Yeap HL, Axford JK, Popovici J, Endersby NM, Iturbe-Ormaetxe I, Ritchie SA, et al. Assessing quality of life-shortening Wolbachia-infected Aedes aegypti mosquitoes in the field based on capture rates and morphometric assessments. Parasites & vectors. 2014;7(1):1-13.

17. Kambris Z, Cook PE, Phuc HK, Sinkins SP. Immune activation by life-shortening Wolbachia and reduced filarial competence in mosquitoes. Science. 2009;326(5949):134-6.

18. Dutra HLC, Lopes da Silva V, da Rocha Fernandes M, Logullo C, Maciel-de-Freitas R, Moreira LA. The influence of larval competition on Brazilian Wolbachia-infected Aedes aegypti mosquitoes. Parasites & Vectors. 2016;9(1):1-15.

19. Kho EA, Hugo LE, Lu G, Smith DD, Kay BH. Effects of larval nutrition on Wolbachia-based dengue virus interference in Aedes aegypti (Diptera: Culicidae). Journal of medical entomology. 2016;53(4):894-901.

20. Turley AP, Smallegange RC, Takken W, Zalucki MP, O'NEILL SL, McGraw EA. Wolbachia infection does not alter attraction of the mosquito Aedes (Stegomyia) aegypti to human odours. Medical and veterinary entomology. 2014;28(4):457-60.

21. Beebe NW, Pagendam D, Trewin BJ, Boomer A, Bradford M, Ford A, et al. Releasing incompatible males drives strong suppression across populations of wild and Wolbachia-carrying Aedes aegypti in Australia. Proceedings of the National Academy of Sciences. 2021;118(41):e2106828118.

22. Bian G, Xu Y, Lu P, Xie Y, Xi Z. The endosymbiotic bacterium Wolbachia induces resistance to dengue virus in Aedes aegypti. PLoS pathogens. 2010;6(4):e1000833.

23. de Oliveira S, Villela DAM, Dias FBS, Moreira LA, Maciel de Freitas R. How does competition among wild type mosquitoes influence the performance of Aedes aegypti and dissemination of Wolbachia pipientis? PLoS Neglected Tropical Diseases. 2017;11(10):e0005947.

24. Caragata EP, Rezende FO, Simões TC, Moreira LA. Diet-induced nutritional stress and pathogen interference in Wolbachia-infected Aedes aegypti. PLoS neglected tropical diseases. 2016;10(11):e0005158.

25. Geoghegan V, Stainton K, Rainey SM, Ant TH, Dowle AA, Larson T, et al. Perturbed cholesterol and vesicular trafficking associated with dengue blocking in Wolbachia-infected Aedes aegypti cells. Nature communications. 2017;8(1):526.

26. Hussain M, Lu G, Torres S, Edmonds JH, Kay BH, Khromykh AA, et al. Effect of Wolbachia on replication of West Nile virus in a mosquito cell line and adult mosquitoes. Journal of virology. 2013;87(2):851-8.

27. Liew C, Soh LT, Chen I, Ng LC. Public sentiments towards the use of Wolbachia-Aedes technology in Singapore. BMC Public Health. 2021;21(1):1-12.

28. Aliota MT, Walker EC, Uribe Yepes A, Dario Velez I, Christensen BM, Osorio JE. The w Mel strain of Wolbachia reduces transmission of chikungunya virus in Aedes aegypti. PLoS neglected tropical diseases. 2016;10(4):e0004677.

29. McMeniman CJ, Lane RV, Cass BN, Fong AW, Sidhu M, Wang Y-F, et al. Stable introduction of a life-shortening Wolbachia infection into the mosquito Aedes aegypti. Science. 2009;323(5910):141-4.

30. Moreira LA, Saig E, Turley AP, Ribeiro JM, O'Neill SL, McGraw EA. Human probing behavior of Aedes aegypti when infected with a life-shortening strain of Wolbachia. PLoS neglected tropical diseases. 2009;3(12):e568.

31. Aliota MT, Peinado SA, Velez ID, Osorio JE. The w Mel strain of Wolbachia reduces transmission of Zika virus by Aedes aegypti. Scientific reports. 2016;6(1):28792.

32. Quek S, Cook DA, Wu Y, Marriott AE, Steven A, Johnston KL, et al. Wolbachia depletion blocks transmission of lymphatic filariasis by preventing chitinase-dependent parasite exsheathment. Proceedings of the National Academy of Sciences. 2022;119(15):e2120003119.

33. Waymire E, Duddu S, Yared S, Getachew D, Dengela D, Bordenstein SR, et al. Wolbachia 16S rRNA haplotypes detected in wild Anopheles stephensi in eastern Ethiopia. Parasites & Vectors. 2022;15(1):1-11.

34. Chrostek E, Gerth M. Is Anopheles gambiae a natural host of Wolbachia? MBio. 2019;10(3):10.1128/mbio. 00784-19.

35. Niang EHA, Bassene H, Makoundou P, Fenollar F, Weill M, Mediannikov O. First report of natural Wolbachia infection in wild Anopheles funestus population in Senegal. Malaria journal. 2018;17(1):1-6.

36. Baldini F, Segata N, Pompon J, Marcenac P, Robert Shaw W, Dabiré RK, et al. Evidence of natural Wolbachia infections in field populations of Anopheles gambiae. Nature communications. 2014;5(1):3985.

37. Joshi D, McFadden MJ, Bevins D, Zhang F, Xi Z. Wolbachia strain w AlbB confers both fitness costs and benefit on Anopheles stephensi. Parasites & vectors. 2014;7(1):1-9.

38. Joshi D, Pan X, McFadden MJ, Bevins D, Liang X, Lu P, et al. The maternally inheritable Wolbachia w AlbB induces refractoriness to Plasmodium berghei in Anopheles stephensi. Frontiers in microbiology. 2017;8:366.

39. Wong ML, Liew JWK, Wong WK, Pramasivan S, Mohamed Hassan N, Wan Sulaiman WY, et al. Natural Wolbachia infection in field-collected Anopheles and other mosquito species from Malaysia. Parasites & vectors. 2020;13:1-15.

40. Hughes GL, Vega-Rodriguez J, Xue P, Rasgon JL. Wolbachia strain wAlbB enhances infection by the rodent malaria parasite Plasmodium berghei in Anopheles gambiae mosquitoes. Applied and environmental microbiology. 2012;78(5):1491-5.

41. Hughes GL, Koga R, Xue P, Fukatsu T, Rasgon JL. Wolbachia infections are virulent and inhibit the human malaria parasite Plasmodium falciparum in Anopheles gambiae. PLoS pathogens. 2011;7(5):e1002043.

42. Gomes FM, Hixson BL, Tyner MD, Ramirez JL, Canepa GE, Alves e Silva TL, et al. Effect of naturally occurring Wolbachia in Anopheles gambiae sl mosquitoes from Mali on Plasmodium falciparum malaria transmission. Proceedings of the National Academy of Sciences. 2017;114(47):12566-71.

43. Straub TJ, Shaw WR, Marcenac P, Sawadogo SP, Dabiré RK, Diabaté A, et al. The Anopheles coluzzii microbiome and its interaction with the intracellular parasite Wolbachia. Scientific Reports. 2020;10(1):13847.

44. Shaw WR, Marcenac P, Childs LM, Buckee CO, Baldini F, Sawadogo SP, et al. Wolbachia infections in natural Anopheles populations affect egg laying and negatively correlate with Plasmodium development. Nature communications. 2016;7(1):11772.

45. Baldini F, Rougé J, Kreppel K, Mkandawile G, Mapua SA, Sikulu-Lord M, et al. First report of natural Wolbachia infection in the malaria mosquito Anopheles arabiensis in Tanzania. Parasites & vectors. 2018;11(1):1-7.

46. Karami M, Moosa-Kazemi SH, Oshaghi MA, Vatandoost H, Sedaghat MM, Rajabnia R, et al. Wolbachia endobacteria in natural populations of Culex pipiens of Iran and its phylogenetic congruence. Journal of arthropod-borne diseases. 2016;10(3):347.

47. Bergman A, Hesson JC. Wolbachia prevalence in the vector species Culex pipiens and Culex torrentium in a Sindbis virus-endemic region of Sweden. Parasites & Vectors. 2021;14(1):428.

48. Hague MT, Caldwell CN, Cooper BS. Pervasive effects of Wolbachia on host temperature preference. MBio. 2020;11(5):10.1128/mbio. 01768-20.

49. Glaser RL, Meola MA. The native Wolbachia endosymbionts of Drosophila melanogaster and Culex quinquefasciatus increase host resistance to West Nile virus infection. PloS one. 2010;5(8):e11977.

50. Zhang D, Wang Y, He K, Yang Q, Gong M, Ji M, et al. Wolbachia limits pathogen infections through induction of host innate immune responses. Plos one. 2020;15(2):e0226736.

51. Atyame CM, Delsuc F, Pasteur N, Weill M, Duron O. Diversification of Wolbachia endosymbiont in the Culex pipiens mosquito. Molecular Biology and Evolution. 2011;28(10):2761-72.

52. Duron O, Labbé P, Berticat C, Rousset F, Guillot S, Raymond M, et al. High Wolbachia density correlates with cost of infection for insecticide resistant Culex pipiens mosquitoes. Evolution. 2006;60(2):303-14.

53. Echaubard P, Duron O, Agnew P, Sidobre C, Noël V, Weill M, et al. Rapid evolution of Wolbachia density in insecticide resistant Culex pipiens. Heredity. 2010;104(1):15-9.

54. Berticat C, Rousset F, Raymond M, Berthomieu A, Weill M. High Wolbachia density in insecticide–resistant mosquitoes. Proceedings of the Royal Society of London Series B: Biological Sciences. 2002;269(1498):1413-6.

55. Shemshadian A, Vatandoost H, Oshaghi MA, Abai MR, Djadid ND. Relationship between Wolbachia infection in Culex quinquefasciatus and its resistance to insecticide. Heliyon. 2021;7(4).

56. Díaz-Nieto LM, Gil MF, Lazarte JN, Perotti MA, Berón CM. Culex quinquefasciatus carrying Wolbachia is less susceptible to entomopathogenic bacteria. Scientific Reports. 2021;11(1):1094.
